# Integrated Serum Pharmacochemistry and Network Pharmacology Approach to Explore the Effective Components and Potential Mechanisms of Menispermi Rhizoma Against Myocardial Ischemia

**DOI:** 10.3389/fchem.2022.869972

**Published:** 2022-05-19

**Authors:** Jinxia Wei, Yingying Yu, Yue Zhang, Lingzhi Li, Xia Li, Jia Shao, Yubo Li

**Affiliations:** ^1^ School of Chinese Materia Medica, Tianjin University of Traditional Chinese Medicine, Tianjin, China; ^2^ Department of Pharmacy, Logistical University of Chinese People’s Armed Police, Tianjin, China; ^3^ Department of Health Service, Hunan Provincial Hospital of Chinese People’s Armed Police, Changsha, China; ^4^ Department of Pharmacy, Tianjin First Central Hospital, Tianjin, China

**Keywords:** Menispermi Rhizoma, myocardial ischemia, serum pharmacochemistry, network pharmacology, molecular docking

## Abstract

**Background:** Myocardial ischemia (MI) is a leading cause of death worldwide. Menispermi Rhizoma is a traditional Chinese medicine that exerts a variety of beneficial pharmacological activities in many diseases, including MI.

**Purpose:** Serum pharmacochemistry and network pharmacology were used to explore the material basis and mechanism of action of Menispermi Rhizoma against MI.

**Methods:** The absorbed components of Menispermi Rhizoma in rat plasma were analyzed by ultra-performance liquid chromatography quadrupole time-of-flight tandem mass spectrometry (UPLC-Q-TOF/MS). The key components, targets, pathways, and interrelated information were obtained by network pharmacology. The potential effective components of Menispermi Rhizoma against MI were screened by methyl-thiazolyl-tetrazolium (MTT) assay, and the cardioprotective effect and mechanism of active components were verified by Western blotting and molecular docking.

**Results:** In total, 25 absorbed components of Menispermi Rhizoma in plasma were identified. Network pharmacology revealed 81 major targets of Menispermi Rhizoma against MI, mainly involving the regulation of the PI3K/AKT and MAPK pathways. *In vitro* validation of H9c2 cells revealed that acutumine, daurisoline, dauricoside, and 6-*O*-demethylmenisporphine are the main bioactive components of Menispermi Rhizoma. The levels of lactate dehydrogenase, creatine kinase, and malondialdehyde (MDA) were significantly decreased by four alkaloids, whereas the activities of superoxide dismutase (SOD) and glutathione (GSH) were significantly increased. Four alkaloids effectively protected H9c2 cells against OGD-induced apoptosis by Hoechst/PI staining and flow cytometry assay. Western blotting results showed that the four alkaloids upregulated the expression ratio of Bcl-2/Bax and downregulated the expression levels of Cyt-C and cleaved caspase 3, which further supported the anti-cardiomyocyte apoptosis and antioxidative stress effect of Menispermi Rhizoma. Molecular docking confirmed that the four compounds were capable of binding to AKT1, MAPK1, EGFR, CASP3, and MAPK8 proteins, suggesting the protective effect of Menispermi Rhizoma on MI *via* PI3K/AKT, MAPK, and apoptosis pathways.

**Conclusion:** Menispermi Rhizoma exerted cardioprotective effects through the effect characteristics: multiple-ingredient, multi-target, and multi-pathway. This research provided a reference for further mechanistic research on wider applications of Menispermi Rhizoma for MI treatment.

## 1 Introduction

Myocardial ischemia (MI) is a pathological condition in which blood perfusion in the heart is reduced, leading to a decrease in the oxygen supply to the heart and abnormal metabolism of myocardial energy, which cannot support the normal operation of the heart (Heusch, 2016). Despite recent improvements in living standards, the prevalence of myocardial infarction continues to increase annually. MI has become a common and frequently occurring disease in middle-aged and elderly individuals (Han et al., 2015; Wang et al., 2019; Fan et al., 2020). The etiology of MI is complex and diverse, wherein coronary atherosclerotic heart disease (coronary heart disease) is the most common and primary cause. MI can cause sudden death and myocardial infarction, among other adverse events (Hasan et al., 2020). At present, drugs to relieve symptoms and improve ischemia mainly include beta-receptor blockers, nitrates, and calcium channel blockers, which are used to prevent and alleviate MI. However, owing to its complex etiology, the efficacy of these drugs in the treatment of MI remains limited. In addition, these drugs can cause serious adverse reactions, such as central nervous system injury, digestive system injury, kidney injury, and hypotension (Sica, 2006; Münzel and Gori, 2013; do Vale et al., 2019).

*Menispermi dauricum* DC. is a plant of the Menispermaceae family, which is widely distributed in China. The rhizome of this plant is used in traditional Chinese medicine (TCM) and is known as Menispermi Rhizoma (“BeiDouGen” in Chinese) (Wei et al., 2021). Menispermi Rhizoma has a wide range of pharmacological activities, such as anti-hypoxia (Shao et al., 2019), anti-inflammatory (Sun et al., 2014; Qiao et al., 2019), anti-Alzheimer’s disease (Liu et al., 2018), anti-arrhythmia (Liu and Han, 2000), and anti-MI effects (Yu et al., 2019). The extensive pharmacological activities could be attributed to the abundant bioactive compounds in this herb. Alkaloids are major active constituents of Menispermi Rhizoma. It was reported that the phenolic alkaloids of Menispermi Rhizoma could attenuate myocardial–cerebral ischemia/reperfusion injury by regulating the generation of reactive oxygen species (Zhao et al., 2012) and enhancing the activity of superoxide dismutase (SOD) (Wang et al., 2005), which verify that Menispermi Rhizoma is involved in the regulation of oxidative stress in myocardial injury. In an MI–reperfusion injury model of rabbits, phenolic alkaloids decreased the content of malondialdehyde (MDA) and increased the activity of SOD in the serum, indicating that the phenolic alkaloids of Menispermi Rhizoma can reduce lipid peroxidation injury, thus playing a protective role in MI–reperfusion injury (Wang et al., 2001). In addition, the phenolic alkaloids of Menispermi Rhizoma can induce the expression of Bcl-2; inhibit Bax, caspase 3 protein, and caspase 3 mRNA expression; and reduce the neuronal apoptosis rate in cerebral ischemia rats (Guo et al., 2015). Hence, the anti-ischemic activity of phenolic alkaloids is closely related to antioxidative stress and antiapoptotic effects. The phenolic alkaloids from Menispermi Rhizoma are a mixture of fat-soluble alkaloids. Although there have been many reports on the anti-MI effects of phenolic alkaloids of Menispermi Rhizoma, the material basis and molecular mechanism of Menispermi Rhizoma for the prevention and treatment of MI remain unclear.

Serum pharmacochemistry of TCM is designed to screen the pharmacodynamic material basis of TCM from the constituents absorbed into the blood after oral administration. The theory and method are in accordance with the effect characteristics of TCMs and reflect the interaction between the body and the drugs. Currently, serum pharmacochemistry has become an effective pathway for researching the material basis of TCM efficacy, which has been recognized and used widely (Yan et al., 2015). Network pharmacology is a branch of pharmacology, which uses network methods to analyze the multi-component, multi-target, and multi-pathway synergistic relationship between drugs, diseases, and targets. The fundamental idea of network pharmacology has much in common with the holistic view of TCM. It enables researchers to fully understand the efficacy and mechanism of multiple components in TCM from a holistic perspective. It has been widely used in the field of TCM modern research (Li F.-H. et al., 2021).

**FIGURE 1:**
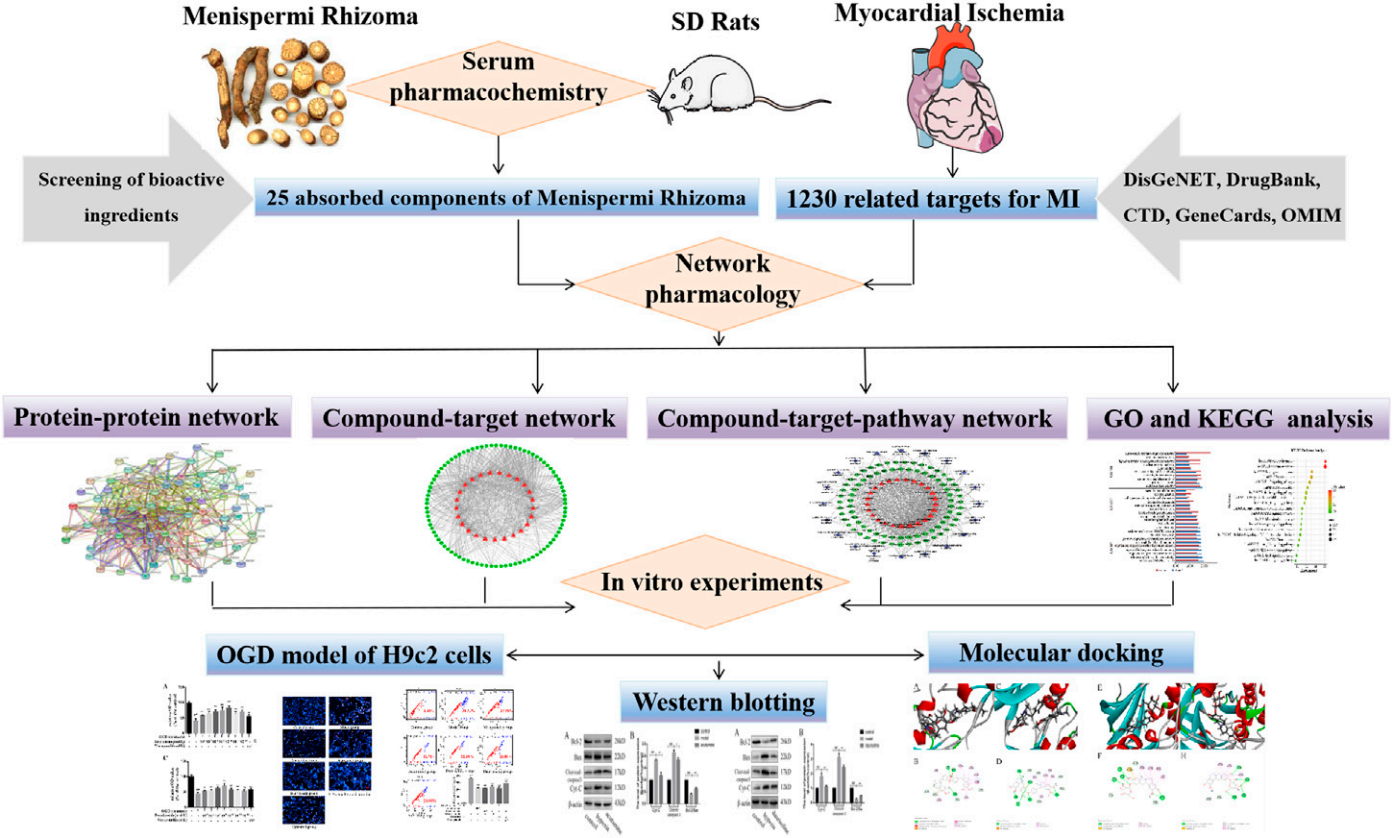
Technological roadmap.

In the present study, we integrated serum pharmacochemistry and network pharmacology to reveal the potential active components and mechanism of Menispermi Rhizoma on MI. The absorbed components of Menispermi Rhizoma in rat plasma were identified and selected for the network pharmacology analysis. Then, the potential active components and signaling pathways screened by network pharmacology were verified by *in vitro* experiments. The protective effect of active components of Menispermi Rhizoma on oxygen-glucose deprivation (OGD)-injured H9c2 cells was assayed by methyl-thiazolyl-tetrazolium (MTT) method. The indexes of oxidative stress and apoptosis rates were detected. The expression of key proteins in the mitochondrion apoptosis pathway, such as Cyt-C, cleaved caspase 3, Bax, and Bcl-2, was examined by Western blotting. Meanwhile, the key proteins with a high association with MI were confirmed by molecular docking. Overall, this study attempts to predict and confirm the potential active components and mechanisms of Menispermi Rhizoma against MI. Figure 1 shows a technological roadmap.

## 2 Materials and Methods

### 2.1 Chemicals and Reagents

Menispermi Rhizoma was purchased from a Yuntian traditional Chinese medicine store, Anguo city (Hebei, China). Acutumine, daurisoline, dauricoside, and 6-*O*-demethylmenisporphine (purity ≥98%) were isolated, purified, and characterized in our laboratory (Shao et al., 2019). HPLC-grade methanol, acetonitrile, and formic acid were obtained from Thermo Fisher Scientific (United States). An H1750R centrifuge (Changsha High Tech Industrial Development Zone Xiangyi Centrifuge Instrument Co., Ltd.), XW-80A vortex mixer (Haimen Kylin-Bell Lab Instruments Co., Ltd.), and a nitrogen blowing instrument (Shanghai Jingfu Instrument Co., Ltd.) were used. Sprague–Dawley (SD) rats (SPF grade) were purchased from the Beijing Military Medical Science Academy of the PLA (Beijing, China). MTT was purchased from Solarbio Co., Ltd. (Beijing, China). Fetal cardiomyocyte-derived H9c2 cells (ATCC CRL-1446) were purchased from the Chinese Typical Culture Preservation Center at Wuhan University. Lactate dehydrogenase (LDH), creatine kinase (CK), SOD, glutathione (GSH), and MDA kits were purchased from Nanjing Jiancheng Bioengineering institute (Nanjing, China). A bicinchoninic acid (BCA) quantitative kit was acquired from ComWin Biotech Co., Ltd. (Beijing, China). The Hoechst/PI staining kit was purchased from Solarbio Co., Ltd (Beijing, China), and the YF488-annexin V/PI cell apoptosis assay kit was obtained from US Everbright® Inc. (Suzhou, China). Antibodies were obtained from Bioss Antibodies (Beijing, China), including cytochrome C (Cyt-C, 1:800), B-cell lymphoma 2 (Bcl-2, 1:800), Bcl-2-associated X protein (Bax, 1:800), and cleaved caspase 3 (1:800). Horseradish peroxidase (HRP)-labeled goat anti-rabbit IgG (1:5000) and goat anti-mouse IgG antibodies (1:5000) were obtained from Abcam (Cambridge, MA, United States).

### 2.2 Animal Experiments

#### 2.2.1 Preparation of the Menispermi Rhizoma Extract

For the experiment, 50 g of Menispermi Rhizoma was weighed, mixed with 20 times 95% ethanol, and refluxed twice for 1 h each time. The resulting extract was filtered, and the filter residue was refluxed twice with 20 times 75% ethanol for 1 h each time. The filtrates were combined and concentrated under reduced pressure to obtain the ethanol extract. To prepare 0.333 g/ml suspension of extract, 3.84 g of ethanol extract of Menispermi Rhizoma was precisely weighed and 0.5% sodium carboxymethyl cellulose (CMC-Na) aqueous solution was added.

#### 2.2.2 Drug Administration and Sample Collection

The rats were adaptively fed standard feed (American chicken meal, Peruvian fish meal, soybean meal, vegetable oil, bran, corn, flour, compound vitamins, compound trace elements, calcium hydrogen phosphate, stone powder, etc.) for 1 week with a light/dark cycle of 12 h and *ad libitum* access to drinking water. The indoor temperature was set to 22–26°C with a relative humidity of 40–60%. Before the experiment, the rats were fasted for 12 h and allowed to drink freely. This study was approved by the Experimental Animal Ethics Committee of Tianjin University of Traditional Chinese Medicine (approval No. TCM-LAEC2020085). All procedures were in accordance with the Guide for the Care and Use of Laboratory Animals (National Institutes of Health). In total, 12 SD rats were randomly divided into blank and experimental groups. The experimental group was administered the Menispermi Rhizoma extract by gavage at a 2.5 g/kg dose once a day for three consecutive days (Zhang et al., 2021). The blank group was given the same amount of 0.5% CMC-Na aqueous solution according to the same protocol. After the final dose, 0.5 mL blood samples from the eye orbital venous plexus were collected in a centrifuge tube precoated with heparin sodium at designated time points (0.25, 0.75, 1, 3, 5, 7, 8, 10, 12, and 24 h). After centrifugation at 14,000 rpm for 10 min, the supernatant was separated to obtain the drug-containing plasma.

#### 2.2.3 Sample Preparation

The drug-containing plasma from the 10 time points in the experimental group were mixed. The combined plasma sample (1 mL) was added to 4 mL of acetonitrile, vortexed for 3 min, and centrifuged at 14,000 rpm for 10 min. The supernatant was transferred to another clean centrifuge tube and dried under nitrogen flow (35°C). The blood samples in the blank group were combined, and the treatment method was the same as that in the experimental group. The dried residue was dissolved in 200 μL methanol–water solution (1:1), vortexed for 1 min, and centrifuged at 14,000 rpm for 10 min. The resulting supernatant was filtered through a 0.22 μm microporous membrane filter and analyzed.

#### 2.2.4 UPLC-Q-TOF/MS Analysis

Waters Acquity UPLC Class I ultra-performance liquid chromatography (Waters, United States) was performed for the sample detection using a chromatographic column (100 mm × 2.1 mm, 1.7 μm) (ACQUITY UPLC BEH C_18_ Column; Waters, United States) with a temperature of 40°C. The mobile phase consisted of 0.1% formic acid aqueous solution (aqueous phase A) and acetonitrile (organic phase B), using a gradient elution procedure as follows: 0–2 min, 5–8% B; 2–10 min, 8–15% B; 10–15 min, 15–30% B; 15–20 min, 30–80% B; 20–25 min, 80–100% B; 25–27 min, 100–5% B; and 27–30 min, 5% B. The flow rate was 0.3 mL/min, and the injection volume was 2 μL. Q-TOF/MS mass spectrometry was performed using an electrospray ion source (ESI) and a full scan analysis in the positive ion mode. The quality range of the data acquisition was 50–1500, and the parameters of the ion source were as follows: capillary voltage, 3.0 kV; ion source temperature, 120°C; sampling cone voltage, 40 V; cone gas flow, 50 L/h; desolvation gas flow, 800 L/h; desolvation temperature, 325°C; and collision energy, 20–50 eV. High-purity nitrogen served as the auxiliary gas. Data acquisition and processing were performed using Masslynx 4.1 (Waters, United States).

#### 2.2.5 Structural Identification of Absorbed Compounds

UPLC-Q-TOF/MS was used to identify the absorbed compounds. The structures of absorbed prototype compounds were identified according to the reference substances and the MS data reported in the literatures (Sun et al., 2014; Li et al., 2017). Then, the structures of the known metabolites of Menispermi Rhizoma were identified according to the MS experimental data reported in the literature (Wang et al., 2009).

### 2.3 Network Pharmacology Analysis

#### 2.3.1 Target Prediction of Absorbed Components

The SDF files for the 3D structures of absorbed components were downloaded from the PubChem database (https://pubchem.ncbi.nlm.nih.gov/). Unretrieved components were drawn using ChemDraw software and saved in SDF file format. Using the PharmMapper (http://www.lilab-ecust.cn/pharmmapper/) platform, we predicted the potential targets of the compounds. Finally, the predicted target genes of the absorbed components were obtained by merging and deleting duplicate items.

#### 2.3.2 Collection of Potential Targets for Myocardial Ischemia

DisGeNET (https://www.disgenet.org/), DrugBank (https://www.drugbank.ca/), OMIM (https://omim.org/), GeneCards (https://www.genecards.org/), and CTD (https://ctdbase.org/) databases were used to search for genes related to MI using the keyword “myocardial ischemia”. Using the Venn 2.1.0 online platform (http://bioinformatics.psb.ugent.be/webtools/Venn/), the potential target genes of the absorbed components for the treatment of MI were obtained.

#### 2.3.3 Construction of the Protein–Protein Interaction Network

The potential target gene list obtained from “Section 2.3.2” was imported into the STRING database (https://string-db.org/) to construct the relationship between the targets and obtain the data of the protein–protein interaction (PPI) network with the species set as “*Homo sapiens*”. Node connectivity (degree) was calculated by using the “Network Analyzer” function of Cytoscape 3.7.1. Finally, these network topology attributes were used to screen the key target proteins in the PPI network.

#### 2.3.4 Gene Ontology and Kyoto Encyclopedia of Genes and Genomes Pathway Enrichment Analyses

Gene Ontology (GO) and Kyoto Encyclopedia of Genes and Genomes (KEGG) pathway analyses were performed using the Metascape database (https://metascape.org/gp/index.html#/main/step1), and the results were obtained by taking *p* < 0.01 as the screening condition. Excel was used to draw a bar chart of the GO function enrichment analysis. The results of the KEGG pathway enrichment analysis were input into the Bioinformatics platform (http://www.bioinformatics.com.cn/) to obtain the bubble chart.

#### 2.3.5 Construction of Component–Target–Pathway Network

Taking absorbed components, common targets, and signaling pathways as nodes, the corresponding relationships were established in Excel. The network of the “component–target–pathway” of Menispermi Rhizoma for the treatment of MI was constructed using Cytoscape 3.7.1.

### 2.4 Validation of Compounds by *In Vitro* Experiments

An OGD model of the H9c2 cells was established. The effect of acutumine, daurisoline, dauricoside, and 6-*O*-demethylmenisporphine on the viability of OGD-injured H9c2 cells was detected by an MTT assay. The indexes of LDH, CK, SOD, GSH, and MDA were measured using the assay kit following the manufacturer’s instructions. Hoechst-33342/PI staining and flow cytometry assay were conducted to evaluate the cell apoptosis. A detailed description of the procedure is provided in the Supplementary Material (“Validation of compounds by *in vitro* experiments”).

### 2.5 Western Blotting Analysis

The H9c2 cells were divided into control, model, and drug groups, and an OGD model of H9c2 cells *in vitro* was established to simulate MI *in vivo*. Bcl-2, Bax, Cyt-C, and cleaved caspase 3 were detected by Western blotting. Finally, the developer was prepared according to the proportion and developed in the imaging system to obtain protein bands. The protein bands were quantified using ImageJ software. A detailed description of the corresponding procedure is provided in the Supplementary Material (“Western blotting analysis”).

### 2.6 Molecular Docking

Molecular docking is a theoretical simulation method for drug design by exploring the interaction between receptors and ligands. It mainly studies the intermolecular interactions and predicts their binding patterns and affinities. The three-dimensional crystal structures of the candidate proteins for binding with the effective compounds of Menispermi Rhizoma were obtained from the Protein Structure Database (PDB) (http://www.pdb.org/). To ensure accuracy and consistency, Discovery Studio (2017 R2 client) software was used to prepare and optimize the proteins and drug molecules. In addition, it is necessary to verify whether the docking procedure and parameters are suitable for the receptor proteins. The results of molecular docking were evaluated according to -CDOCKER-INTERACTION-ENERGY scores, interaction sites, and interaction types.

### 2.7 Statistical Analysis

The experimental data were analyzed using IBM SPSS Statistics software (version 21.0) and the results were expressed as the mean ± standard deviation (SD). An independent sample t-test was used for the comparison between the two groups, and one-way ANOVA was used for comparison between multiple groups using the mean value. The difference was statistically significant if *p* values were <0.05.

## 3 Results

### 3.1 Identification of Absorbed Components of Menispermi Rhizoma in Rat Plasma Based on UPLC-Q-TOF/MS

UPLC-Q-TOF/MS was used to compare and analyze the plasma profiles of blank plasma and actual plasma samples after the oral administration of Menispermi Rhizoma extract. A total of 25 absorbed components were identified in the rat plasma samples, including 22 prototype components and 3 metabolites, which are shown in Table 1. The BPI chromatograms of blank rat plasma and actual plasma samples in the positive ion mode are shown in Supplementary Material (Supplementary Figure S1).

**TABLE 1:** UPLC-Q-TOF/MS data of the identified components in rat plasma after oral administration of Menispermi Rhizoma extract.

NO.	RT/min	[M + H]^+^Mea.	[M + H]^+^cal.	ppm	Formula	Compounds	Alkaloid types	MS/MS fragments (m/z)
1	0.79	930.3974	930.3954	2.14	C_48_H_59_N_5_O_12_S	Dauricine GSH conjugate	Bisbenzylisoquinoline alkaloid metabolite	801.5558, 657.6545, 623.7991
							
2	3.65	476.1944	476.1921	4.83	C_24_H_29_NO_9_	Dauricoside	Protoberberine alkaloid	314.1557, 179.0286, 178.1164
3	4.47	384.1230	384.1214	4.17	C_18_H_22_ClNO_6_	Acutumidine	Morphinane alkaloid	341.1072, 305.1804, 241.0734, 213.1082
4	5.01	330.1724	330.1705	5.75	C_19_H_23_NO_4_	Sinomenine	Morphinane alkaloid	239.1766, 223.1221, 209.0158
5	5.39	398.1380	398.1370	2.51	C_19_H_24_ClNO_6_	Acutumine	Morphinane alkaloid	341.0927, 305.1814, 241.1212, 213.1265
6	5.80	314.1392	314.1392	0.00	C_18_H_19_NO_4_	Pessoine	Protoberberine alkaloid	314.2014, 298.9214, 283.2948, 178.1161, 163.0928, 135.0951
7	6.32	300.1609	300.1600	3.00	C_18_H_21_NO_3_	*N*-Methylcoclaurine	Isoquinoline alkaloid	269.1437, 237.1189, 107.0784
8	7.05	328.1544	328.1549	-1.52	C_19_H_21_NO_4_	Stepholidine	Protoberberine alkaloid	178.1148, 163.1256, 151.0217
9	8.36	342.1691	342.1705	-4.09	C_20_H_23_NO_4_	Isocorydine	Aporphine alkaloid	297.1521, 282.1384, 254.1448
10	8.58	597.2988	597.2965	3.85	C_36_H_40_N_2_O_6_	Dauricicoline	Bisbenzylisoquinoline alkaloid	566.2622, 554.2621, 405.1934, 192.1318
11	9.28	314.1786	314.1756	9.55	C_19_H_23_NO_3_	Armepavine	Isoquinoline alkaloid	283.1512, 107.0765
12	9.59	342.1744	342.1705	11.40	C_20_H_24_NO_4_^+^	Magnoflorine	Aporphine alkaloid	311.1394, 297.0578, 265.1034, 237.1346
13	10.46	208.0969	208.0974	-2.40	C_11_H_13_NO_3_	Thalifoline	Isoquinoline alkaloid	151.1125, 119.9165, 91.0816
14	11.13	611.3099	611.3121	-3.60	C_37_H_42_N_2_O_6_	Daurinoline	Bisbenzylisoquinoline alkaloid	594.2781, 580.2714, 566.2524, 206.1465, 192.1308
15	11.21	356.1839	356.1862	-6.46	C_21_H_26_NO_4_^+^	Menisperine	Aporphine alkaloid	311.1436, 279.1290, 264.9904, 236.1970
16	11.46	611.3112	611.3121	-1.47	C_37_H_42_N_2_O_6_	Daurisoline	Bisbenzylisoquinoline alkaloid	568.2858, 192.1262
17	12.21	611.3144	611.3121	3.76	C_37_H_42_N_2_O_6_	Dauricinoline	Bisbenzylisoquinoline alkaloid	580.2327, 388.7544, 192.1297
18	12.63	625.3299	625.3278	3.36	C_38_H_44_N_2_O_6_	Dauricine	Bisbenzylisoquinoline alkaloid	582.3157, 551.2172, 206.1444
19	12.85	611.3137	611.3121	2.62	C_37_H_42_N_2_O_6_	2-*N*-demethyl dauricine	Bisbenzylisoquinoline alkaloid metabolite	580.2396, 568.8126, 552.3140, 420.5792, 206.1308, 192.1282
20	12.89	611.3125	611.3121	0.65	C_37_H_42_N_2_O_6_	2′-*N*-demethyl dauricine	Bisbenzylisoquinoline alkaloid metabolite	594.2560, 580.9526, 566.0503, 206.1457, 190.0058
21	14.07	222.1136	222.1130	2.70	C_12_H_15_NO_3_	*N*-methylcorydaldine	Isoquinoline alkaloid	165.0998, 150.0788, 134.1224
22	18.34	308.0931	308.0923	2.60	C_18_H_13_NO_4_	6-*O*-demethylmenisporphine	Oxoisoaporphine alkaloid	265.1375, 264.2951, 236.1989, 235.1990
23	19.09	292.0990	292.0974	5.48	C_18_H_13_NO_3_	Bianfugecine	Oxoisoaporphine alkaloid	249.1076, 248.1947, 220.1238
24	19.16	352.1184	352.1185	-0.28	C_20_H_17_NO_5_	Dauriporphine/bianfugenine	Oxoisoaporphine alkaloid	322.2847, 308.1107, 294.1447, 251.1216
25	22.40	338.1021	338.1028	-2.07	C_19_H_15_NO_5_	Dauriporphinoline	Oxoisoaporphine alkaloid	322.1305, 294.1348, 251.0741

### 3.2 Protein–Protein Interaction Network Analysis

**FIGURE 2:**
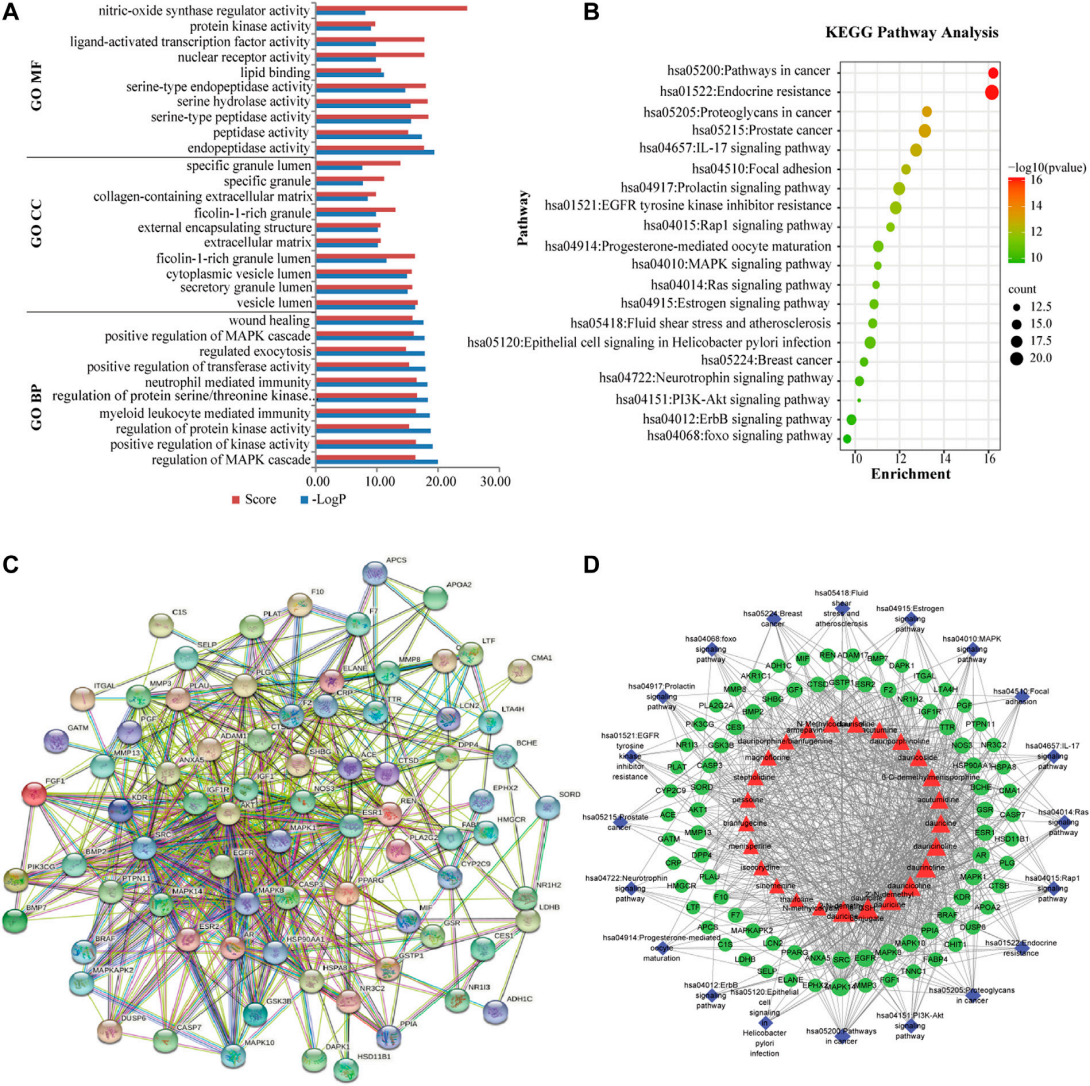
**(A)** Bar chart of GO enrichment analysis. **(B)** Bubble diagram of the KEGG pathway enrichment analysis. **(C)** PPI diagram of common targets. **(D)** Diagram of the “component–target–pathway” network. Red indicates the components, green indicates the common targets, and blue indicates the signaling pathways.

A list of the top 10 targets and their degree values in the PPI network is provided in Table 2. In the PPI network, the target proteins were mainly focused on the pathways related to apoptosis and PI3K/AKT, MAPK, such as AKT1, MAPK1, EGFR, CASP3, IGF1, MAPK8, SRC, and ESR1. These target proteins were closely related to the treatment of MI by Menispermi Rhizoma (Figure 2C).

**TABLE 2:** Top 10 targets of the degree in the PPI network.

Target	Protein name	Uniprot ID	Degree
AKT1	RAC-alpha serine/threonine-protein kinase	P31749	51
MAPK1	Mitogen-activated protein kinase 1	P28482	45
EGFR	Epidermal growth factor receptor	P00533	38
CASP3	Caspase-3	P42574	36
MAPK8	Mitogen-activated protein kinase 8	P45983	36
IGF1	Insulin-like growth factor I	P05019	36
CRP	C-reactive protein	P02741	34
SRC	Proto-oncogene tyrosine-protein kinase Src	P12931	34
PLG	Plasminogen	P42574	31
ESR1	Estrogen receptor	P12931	30

### 3.3 Gene Ontology and Kyoto Encyclopedia of Genes and Genomes Pathway Enrichment Analysis

The GO enrichment analysis showed that the biological processes related to the treatment of MI were mainly involved in the regulation of the MAPK cascade, positive regulation of kinase activity, and regulation of protein kinase activity. In terms of cellular components, the vesicle lumen, secretory granule lumen, and cytoplasmic vesicle lumen were mainly involved, whereas in terms of molecular functions, the treatment of MI was mainly affected by endopeptidase activity, peptidase activity, and serine-type peptidase activity (Figure 2A). The results of the KEGG pathway enrichment analysis showed that the pathways were closely related to the IL-17, RAS, MAPK, PI3K/AKT, and fluid shear stress signaling pathways (Figure 2B).

### 3.4 Construction and Analysis of the Component–Target–Pathway Network

A total of 196 targets were predicted by the PharmMapper database, and 1,230 targets of MI were retrieved from the databases, and 81 common targets were obtained after crossing analysis. Using Cytoscape 3.7.1 software, the network diagram of the “component–target–pathway” was constructed. As shown in Figure 2D, red, green, and blue represent the components, common targets, and signaling pathways, respectively. Through a network topology analysis, it was found that acutumine, daurisoline, dauricoside, 6-*O*-demethylmenisporphine, dauricine, and dauricicoline possess high degrees. The results suggested that these alkaloids might be the potential active components of Menispermi Rhizoma for the treatment of MI.

### 3.5 Effect of Effective Components on H9c2 Cell Viability

**FIGURE 3:**
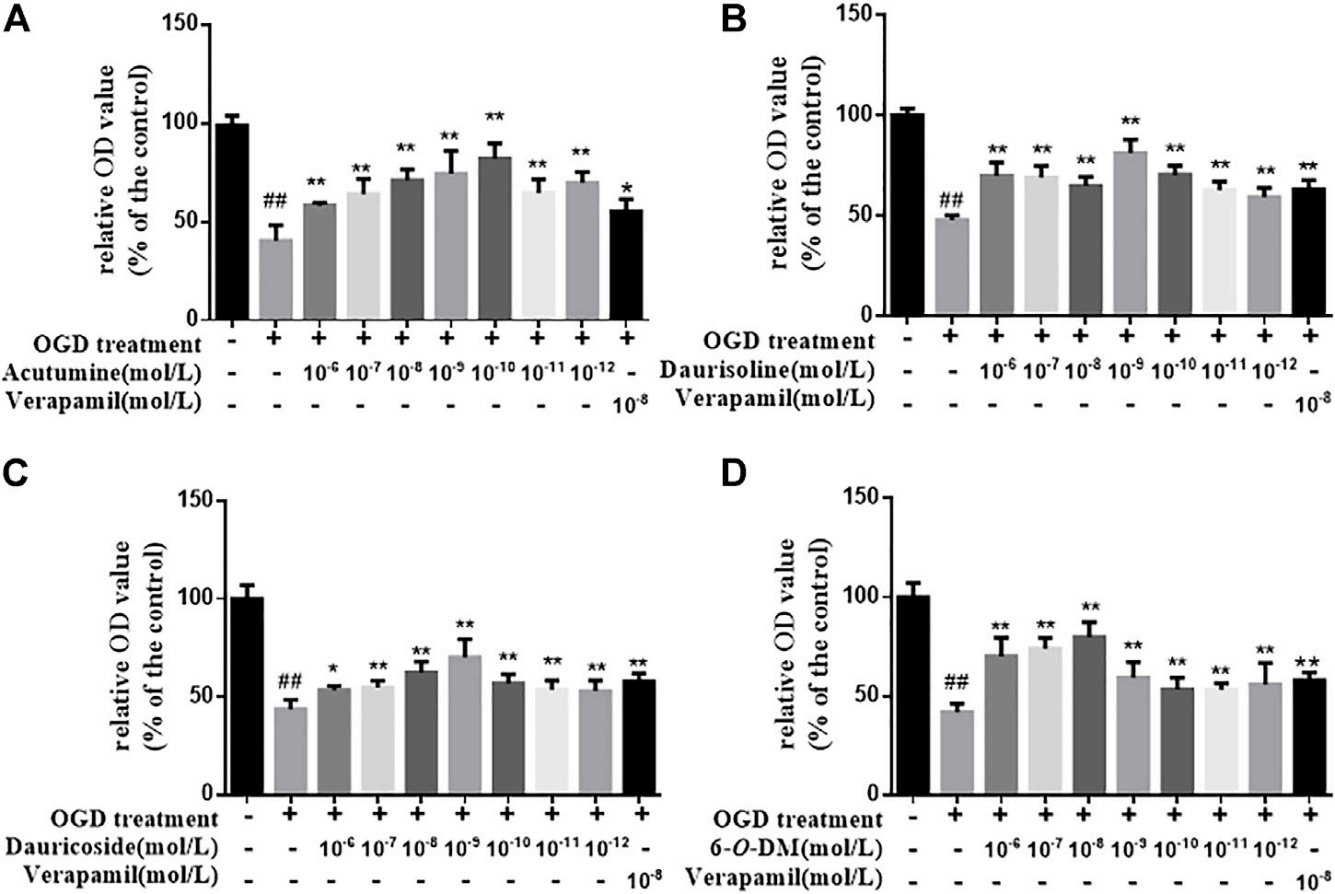
Effects of four alkaloids in different concentrations (10^−12^–10^−6^ mol/L) on the viability of H9c2 cells after OGD treatment for 2 h: **(A)** acutumine, **(B)** daurisoline, **(C)** dauricoside, and **(D)** 6-*O*-demethylmenisporphine (6-*O*-DM). Results are represented as mean ± SD (*n* = 6). ^##^ *p* < 0.01 vs. control group; *p < 0.05, **p < 0.01 vs. model group.

Followed by OGD treatment, the cell viability of the model group decreased significantly compared with the control group (*p* < 0.01). Verapamil (10^−8^ mol/L) was used as the positive control drug. The effects of acutumine, daurisoline, dauricoside, and 6-*O*-demethylmenisporphine in certain concentrations (10^−12^–10^−6^ mol/L) on OGD-injured H9c2 cells are shown in Figure 3. Compared with the model group, the viability of cells treated with acutumine, daurisoline, dauricoside, 6-*O*-demethylmenisporphine, and the positive drug increased significantly (*p* < 0.01), showing that four alkaloids protected H9c2 cells from hypoxia-induced injury in a concentration-dependent manner.

### 3.6 Effect of Four Alkaloids on Myocardial Enzyme Activities and Oxidative Stress Indexes

**FIGURE 4:**
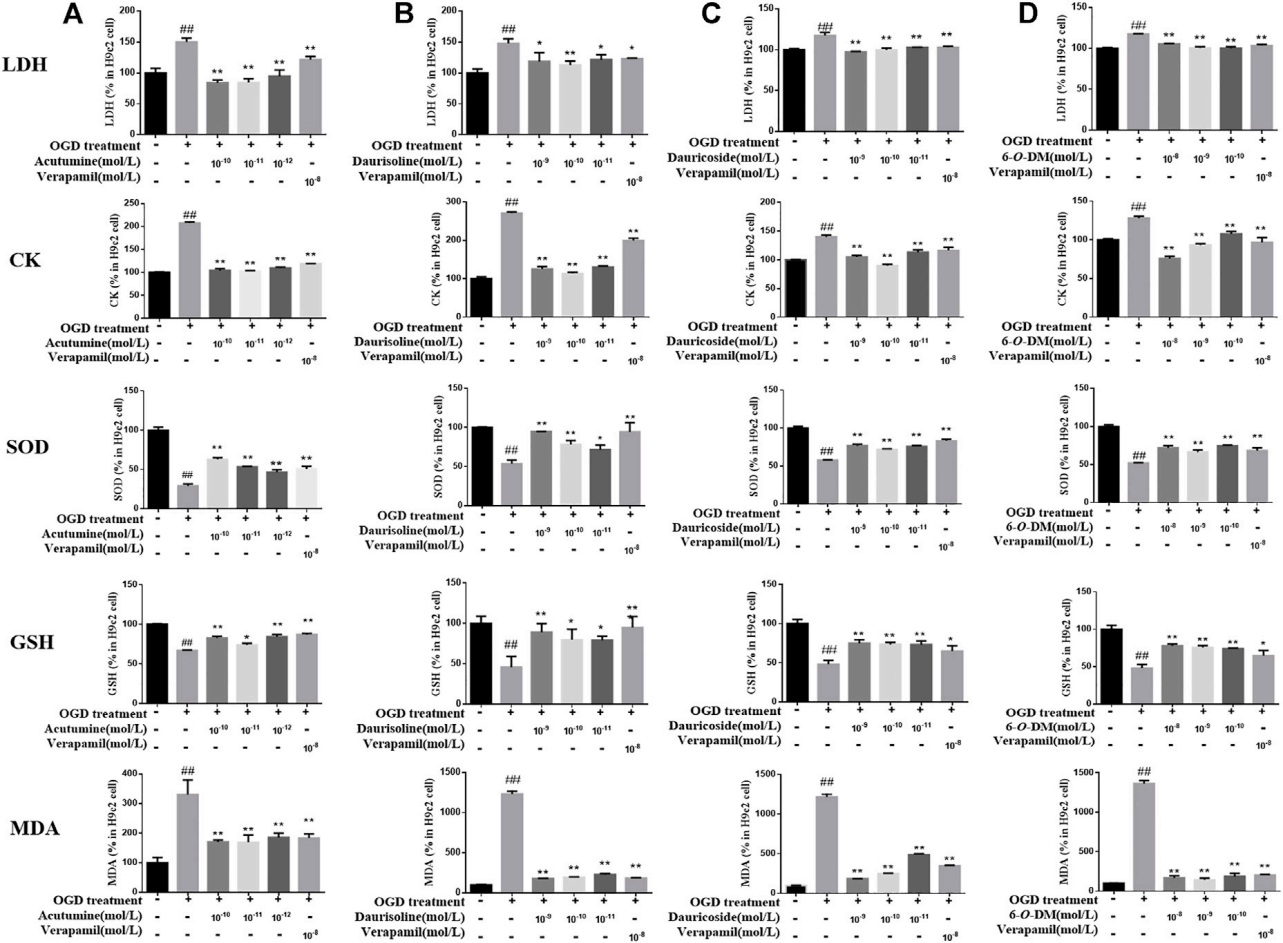
Effects of four alkaloids on the levels of LDH, CK, SOD, GSH, and MDA in OGD-injured H9c2 cells: **(A)** acutumine, **(B)** daurisoline, **(C)** dauricoside, and **(D)** 6-*O*-demethylmenisporphine (6-*O*-DM). Results are represented as mean ± SD (*n* = 3). ^##^ *p <* 0.01 vs. control group; *p < 0.05, **p < 0.01 vs. model group.

LDH leakage and CK release in the culture supernatant were considered the markers of myocardial membrane damage. SOD and GSH are essential for oxygen free radical scavenging. MDA accumulation could result in lipid peroxidation damage to the cell membrane. In order to investigate the protective effects of the four alkaloids on cardiomyocytes against hypoxia-induced oxidative stress damage, the activities of LDH, CK, SOD, GSH, and MDA in different groups were observed. Figure 4 shows the effects of acutumine, daurisoline, dauricoside, and 6-*O*-demethylmenisporphine on the levels of LDH, CK, SOD, GSH, and MDA in OGD-injured H9c2 cells. Compared with the control group, LDH leakage, CK release, and MDA content in the model group increased significantly (*p* < 0.01), while the SOD and GSH activities decreased significantly (*p* < 0.01). Compared with the model group, acutumine, daurisoline, dauricoside, and 6-*O*-demethylmenisporphine decreased the LDH leakage, CK release, and MDA content significantly (*p* < 0.05), and they also increased the SOD activity and GSH content significantly (*p* < 0.05); this was the same as the effect of verapamil on H9c2 cells.

### 3.7 Effect of Four Alkaloids on Cell Apoptosis

**FIGURE 5:**
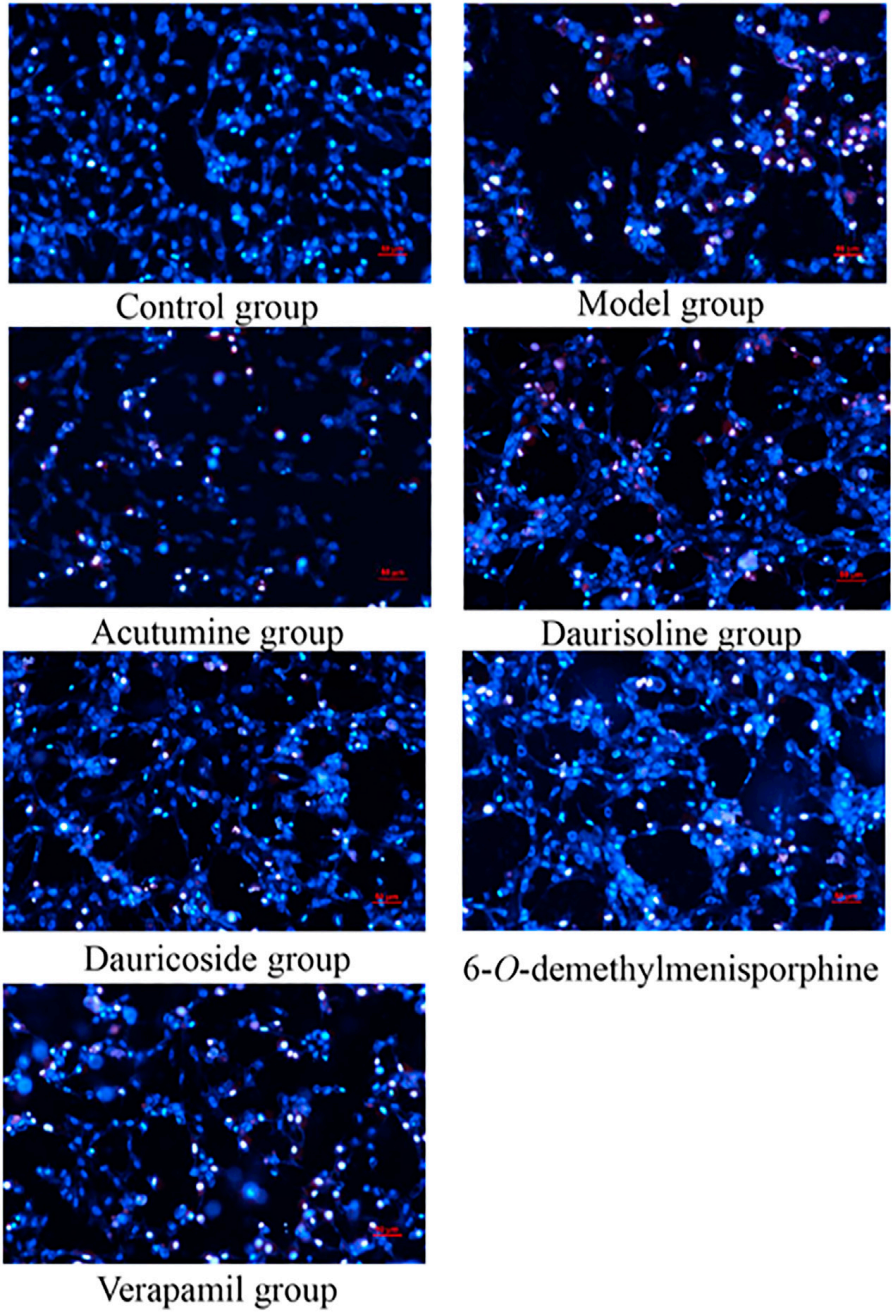
Effects of acutumine, daurisoline, dauricoside, and 6-*O*-demethylmenisporphine, and verapamil on OGD-injured cell apoptosis by Hoechst/PI staining (200×).

Morphological evaluation of apoptotic cardiomyocytes by Hoechst 33342/PI staining: In this study, a Hoechst 33342/PI fluorescent staining kit was used for the morphological evaluation of four alkaloids on injured myocardial cells. After staining with Hoechst/PI, a fluorescent microscope was employed for distinguishing the normal, apoptotic, and necrotic cells. Figure 5 shows the images of Hoechst/PI staining in different groups. The chromatin of normal cells in the control group was evenly dispersed, and the cell membrane was intact, while the apoptotic cells are characterized by nuclear atrophy, chromatin condensation, and fragmentation in the model group. No evident cell apoptosis and necrosis were observed in the control group with a lack of bright fluorescence, while a large number of apoptotic cells with bright blue fluorescence (nuclear condensation and fragmentation) existed in the model group. Similar to the situation, after treatment with drugs, four alkaloids could alleviate severe apoptosis. It has been clarified that less bright blue fluorescence could be observed compared with the model group.

**FIGURE 6:**
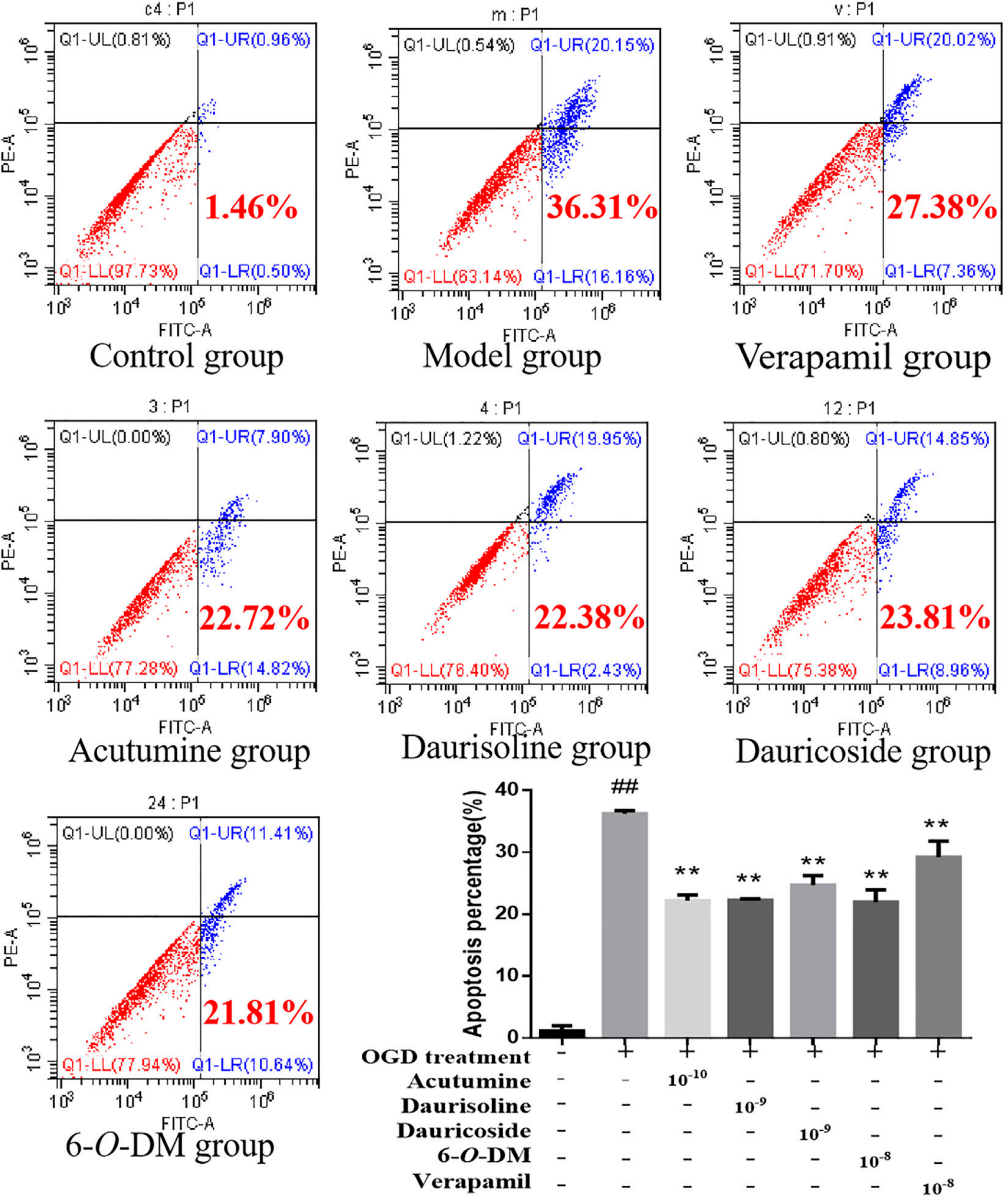
Effects of acutumine, daurisoline, dauricoside, and 6-*O*-demethylmenisporphine (6-*O*-DM), and verapamil on the apoptosis rate of OGD-injured H9c2 cells (Results are represented as mean ± SD (n=3). ^##^ *p* < 0.01 vs. control group; ^##^ *p* < 0.01; vs. model group).

Effects of four alkaloids on H9c2 cell apoptosis rate: Annexin V-FITC/PI double staining was assayed to examine whether the four alkaloids protect the cells against hypoxia-induced apoptosis. The apoptosis rates of H9c2 myocardial cells were measured by flow cytometry, the results of which are shown in Figure 6. The apoptosis rate in the model group was 36.31%, which was significantly higher than that (1.46%) of the control group (*p* < 0.01). Four alkaloids treatment could reduce the apoptosis rate to a lower level compared with the model group (*p* < 0.01), and the apoptosis rates of acutumine, daurisoline, dauricoside, and 6-*O*-demethylmenisporphine groups were 22.72%, 22.38%, 23.81%, and 21.81%, respectively. The results showed that the presence of four alkaloids remarkably inhibited OGD-induced apoptosis in H9c2 cardiomyocytes.

### 3.8 Western Blotting

**FIGURE 7:**
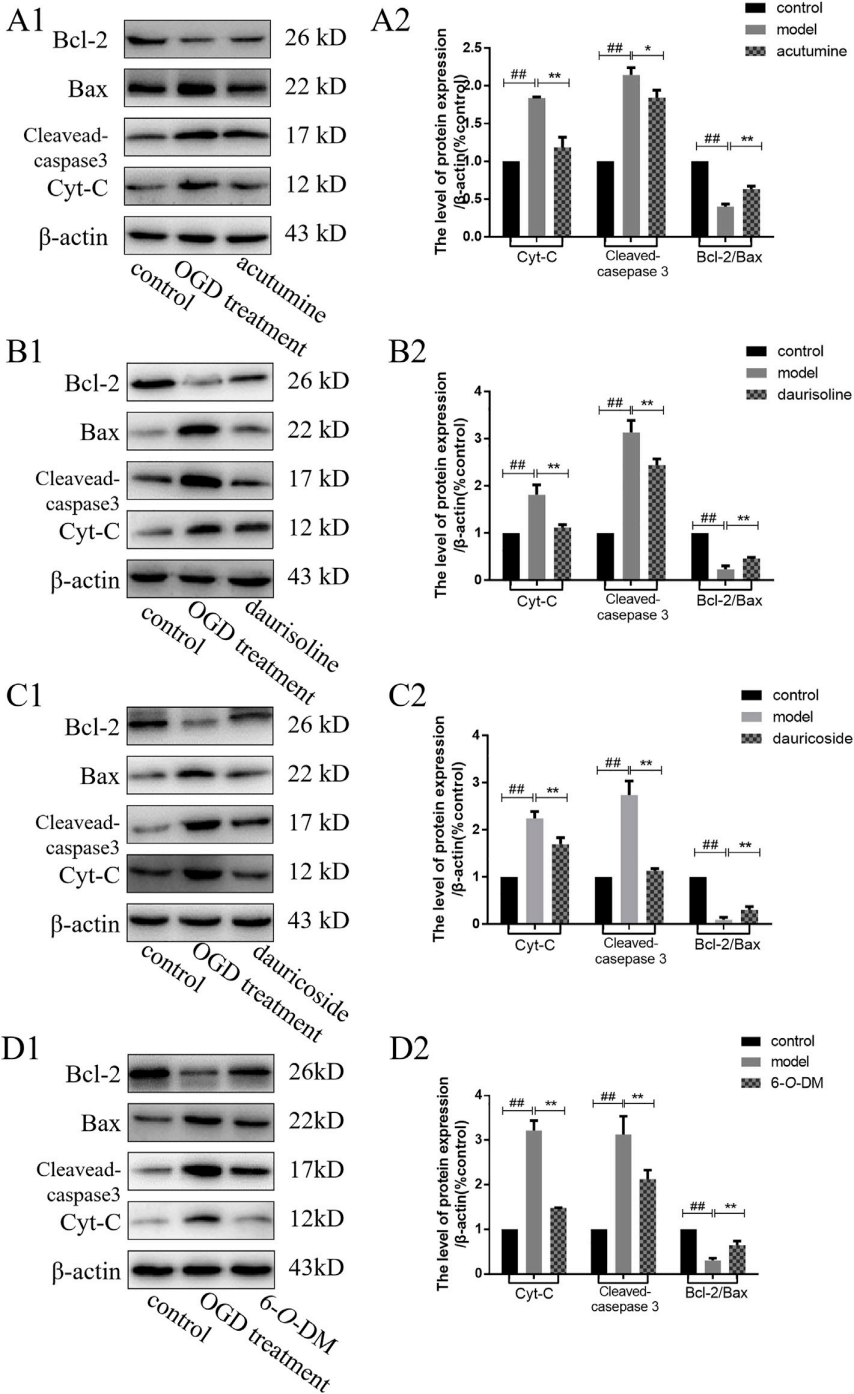
Effects of four alkaloids on the expression levels of mitochondrial apoptosis-related proteins in OGD-injured H9c2 cells: **(A)** acutumine, **(B)** daurisoline, **(C)** dauricoside, and **(D)** 6-*O*-demethylmenisporphine (6-*O*-DM). A1∼D1: The Bcl-2, Bax, cleaved caspase 3, and Cyt-C expressions were examined by Western blotting. A2∼D2: Effects of four alkaloids on protein expression were analyzed. Results are represented as mean ± SD (*n* = 3). ^##^ *p* < 0.01 vs. control group; *p < 0.05, **p < 0.01 vs. model group.

The mechanism of Menispermi Rhizoma against MI was verified by Western blotting analysis. The relative expression of key proteins cleaved caspase 3, Cyt-C, Bcl-2, and Bax were measured and are shown in Figure 7. β-Actin acted as the reference protein. Compared with the control group, the pro-apoptosis proteins Cyt-C and apoptosis executor cleaved caspase 3 were upregulated in the model group (*p* < 0.01). The Bcl-2 family proteins in adjustment were presented in the form of Bcl-2/Bax. Acutumine, daurisoline, dauricoside, and 6-*O*-demethylmenisporphine could regulate the protein expression and participate in the regulation of the mitochondrial apoptosis pathway. After intervention with four alkaloids, the expressions of Cyt-C and cleaved caspase 3 were downregulated, while Bcl-2/Bax was upregulated compared with the model group (*p* < 0.01). The results demonstrated that the mitochondria apoptosis pathway plays an important role in the treatment of MI by Menispermi Rhizoma.

### 3.9 Molecular Docking

To further verify the effective compounds and mechanism of Menispermi Rhizoma for MI treatment, molecular docking studies of the binding of four alkaloids to five target proteins [AKT1 (PDB ID: 6HHG), MAPK1 (PDB ID: 6SLG), EGFR (PDB ID: 3BEL), CASP3 (PDB ID: 1GFW), and MAPK8 (PDB ID: 4G1W)] were performed using the CDocker module in Discovery Studio software. The affinity and binding mode of components and proteins were evaluated according to the docking results. Taking the docking scores (-CDOCKER-INTERACTION-ENERGY) as the judgment standard, the higher the value of -CDOCKER-INTERACTION-ENERGY, the stronger the binding activity of compounds with proteins. The interaction energy scores are presented in Table 3.

**TABLE 3:** Molecular docking results.

Target	PDB ID	Small molecule	-CDOCKER-INTERACTION-ENERGY (kcal/mol)
AKT1	6HHG	Acutumine	49.4816
AKT1	6HHG	Daurisoline	77.1687
AKT1	6HHG	Dauricoside	60.8527
AKT1	6HHG	6-*O*-demethylmenisporphine	49.1930
MAPK1	6SLG	Acutumine	43.0197
MAPK1	6SLG	Daurisoline	71.6839
MAPK1	6SLG	Dauricoside	67.6567
MAPK1	6SLG	6-*O*-demethylmenisporphine	55.8603
EGFR	3BEL	Acutumine	42.8835
EGFR	3BEL	Daurisoline	51.3880
EGFR	3BEL	Dauricoside	63.7087
EGFR	3BEL	6-*O*-demethylmenisporphine	59.6874
CASP3	1GFW	Acutumine	34.5984
CASP3	1GFW	Daurisoline	54.2650
CASP3	1GFW	Dauricoside	46.7455
CASP3	1GFW	6-*O*-demethylmenisporphine	47.7566
MAPK8	4G1W	Acutumine	32.5309
MAPK8	4G1W	Daurisoline	60.2342
MAPK8	4G1W	Dauricoside	48.8537
MAPK8	4G1W	6-*O*-demethylmenisporphine	47.9498

**FIGURE 8:**
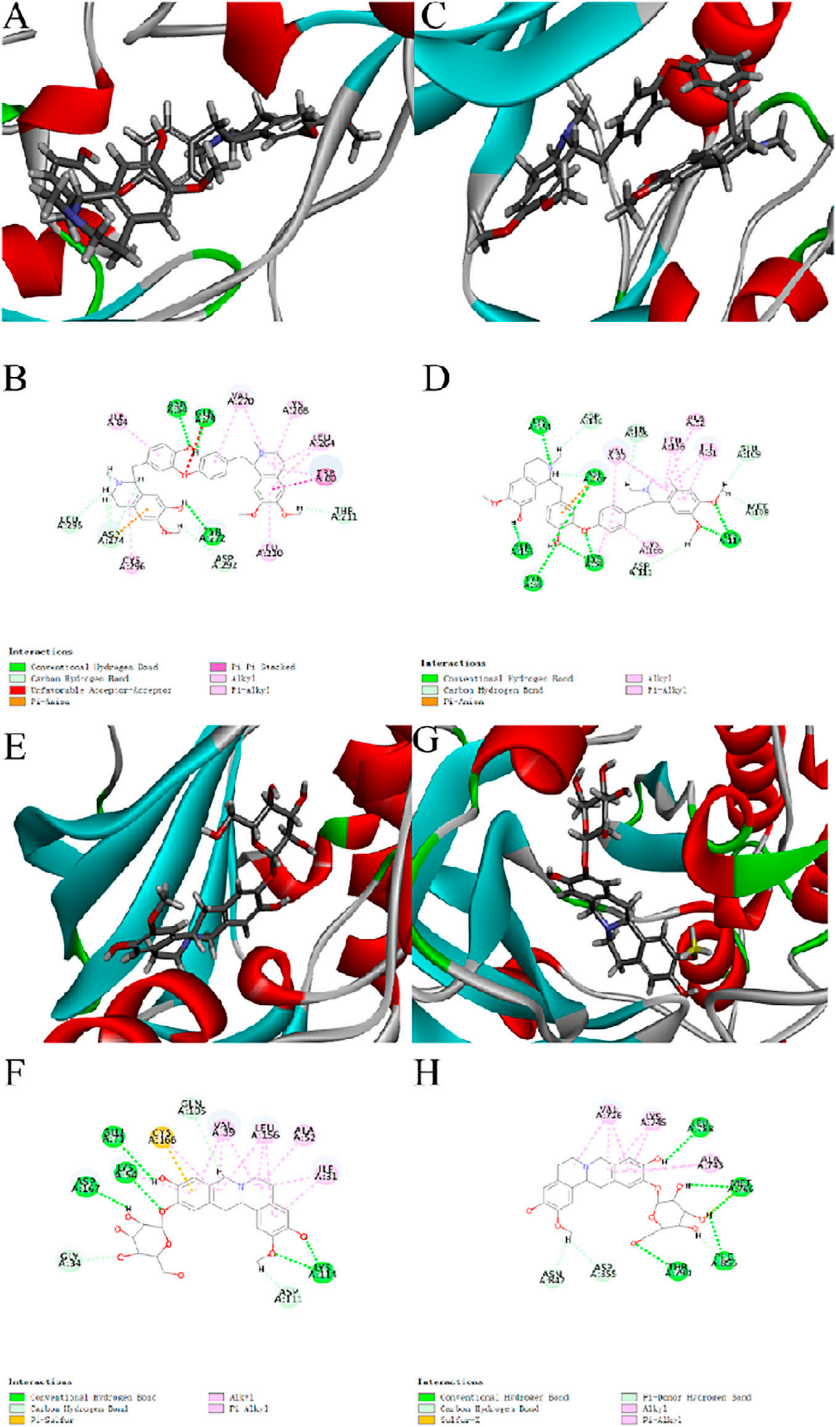
Molecular docking models of daurisoline with AKT1, MAPK1, and dauricoside with MAPK1, EGFR in 3D and 2D diagrams. **(A**,**B)** 3D and 2D diagrams of daurisoline with AKT1. **(C**,**D)** 3D and 2D diagrams of daurisoline and MAPK1. **(E**,**F)** 3D and 2D diagrams of dauricoside and MAPK1. **(G**,**H)** 3D and 2D diagrams of dauricoside and EGFR.

Figure 8 shows the molecular docking models of compounds with proteins in 3D and 2D diagrams. The best docking of the receptor and ligand showed that daurisoline had the highest affinity with AKT1 based on the highest value of -CDOCKER-INTERACTION-ENERGY (77.1687). The receptor–ligand interactions (Figure 8B) primarily included three hydrogen bonds with amino acid residues GLN79, ASN54, and TYR272, and the non-covalent bonds and hydrophobic effects with different amino acid residues (LEU295, ASP274, CYS296, ILE84, ASP292, VAL270, LEU210, THR211, TRP80, LEU264, and LYS268). In addition, daurisoline had good binding activity with MAPK1 protein, while dauricoside had good binding activity with EGFR and MAPK1 proteins. These data (Table 3) suggested that the four alkaloids can be considered as the bioactive components of Menispermi Rhizoma, and they exerted anti-MI effects via MAPK and PI3K/AKT pathways.

## 4 Discussion

TCM is a complex system composed of multiple ingredients, which has the effect characteristics of multi-target and multi-pathway in treating diseases. Owing to the lack of effective research methods, it remains difficult to elucidate the pharmacodynamic substances and mechanism of Menispermi Rhizoma against MI. The inception of network pharmacology comes from the advance in “multi-target, multi-drug” paradigm and opens up a new field for the research of TCM. Currently, network pharmacology has been successfully undertaken to screen bioactive components and reveal the potential pharmacodynamic mechanisms of TCM (Li L. et al., 2021). In this study, we used the UPLC-Q-TOF/MS technology to identify the absorbed components of Menispermi Rhizoma in rat plasma and explore the possible effective components and their mechanisms for MI treatment using network pharmacology. Consistent with previous studies, we found that alkaloids were the main bioactive constituents closely related to the pharmacological and biological properties of Menispermi Rhizoma (Zhou et al., 2018; Qiao et al., 2019). A total of 25 absorbed alkaloids of Menispermi Rhizoma, including prototype components and metabolites, were identified by UPLC-Q-TOF/MS, which belong to various classes, such as bisbenzylisoquinoline, oxoisoaporphine, protoberberine, morphinane, isoquinoline, and aporphine alkaloids. Of 25 absorbed components, 81 potential target genes that are essential for MI treatment were identified. The main targets were involved in several biological functions and processes, such as the MAPK cascade, the positive regulation of kinase activity, and the regulation of protein kinase activity. Through the network topology analysis of PPI and the “component–target–pathway” network, we found that AKT1, MAPK1, EGFR, CASP3, and MAPK8 are key proteins of the absorbed components, and acutumine, daurisoline, dauricoside, 6-*O*-demethylmenisporphine, and dauricine with high degrees were screened out as the potential active components of Menispermi Rhizoma. These key proteins are the core node of the PPI network, involved in the biological processes of cell apoptosis, oxidative stress, inflammation, and immune response in myocardial injury (Sung et al., 2017; Li F.-H. et al., 2021; Li L. et al., 2021). The KEGG pathway enrichment analysis revealed that five signaling pathways were closely related to MI, including the IL-17, RAS, MAPK, PI3K/AKT, and fluid shear stress pathways. These five pathways could be regulated by the absorbed components of Menispermi Rhizoma and mediate a variety of biological processes and cellular responses in the cardiac environment, such as immune regulation, anti-inflammatory, antioxidative stress, and antiapoptosis. Our results suggested that the absorbed components of Menispermi Rhizoma may target multiple key proteins simultaneously and have a functional contribution to the main pathways, leading to the establishment of a new balance in the biological system against MI.

The PI3K/AKT pathway is particularly important in mediating myocardial cell survival, which participates in the regulation of mitochondrial apoptosis. Many studies showed that the activation of the PI3K/AKT pathway is essential for cardioprotective effects (Lin et al., 2018). AKT1 is the core node of the PI3K/AKT signaling pathway and is expressed in almost all tissues, especially high in the brain, heart, and lungs (Du et al., 2014). AKT1 activation has been shown to play a key role in reducing MI injury. MAPK1 and MAPK8 of hub DEGs belong to the MAPK pathway, which can phosphorylate and activate the expression of downstream genes, and regulate cell proliferation, survival, and apoptosis (Jin et al., 2021). The MAPK subfamilies stress kinases, p38 and JNK, can cause inflammation and apoptotic cell death. Many studies have revealed that the inhibition of p38 MAPK reduced cardiomyocyte apoptosis and improved cardiac performance following ischemia–reperfusion injury (Suchal et al., 2016). Apoptosis is an essential contributor to cardiac dysfunction. Caspase 3 is one of the executors of caspases and is related to cell apoptosis. Caspase-dependent apoptosis is considered an important molecular mechanism (Cheng et al., 2018). Bax is a pro-apoptotic molecule, whereas Bcl-2 is an antiapoptosis protein. When the ratio of Bax/Bcl-2 was increased, the permeability of mitochondrial pores increased. Caspase 3 was cleaved into small activated fragments called cleaved caspase 3, and the expression of cleaved caspase 3 may be increased, indicative of an increase in apoptosis (Wang et al., 2020). EGFR is a receptor protein tyrosine kinase (EGFRtk) that can be activated by EGF-like proteins. EGFRtk activity is essential for normal heart development. However, its function in the heart has only recently been demonstrated (Mali et al., 2018). Therefore, we speculated that antiapoptotic effects regulated by MAPK and PI3K/AKT signaling pathways might be the potential mechanisms of Menispermi Rhizoma on MI.

In our previous study, we found that the contents of acutumine, daurisoline, dauricoside, and 6-*O*-demethylmenisporphine were higher not only in Menispermi Rhizoma but also in rat plasma (Wei et al., 2015; Wei et al., 2017; Wei et al., 2020). They were the representative components of morphinane, bisbenzylisoquinoline, protoberberine, and oxoisoaporphine alkaloids, respectively. Hence, acutumine, daurisoline, dauricoside, and 6-*O*-demethylmenisporphine were chosen for the verification of cardioprotective effects and mechanisms predicted by network pharmacology. The effect of the four alkaloids on H9c2 cell viability was detected by MTT assay. After OGD treatment for 2 h, cell viability decreased in the model group, while the proliferation activity of the drug intervention groups significantly increased. When MI or hypoxia occurs, the integrity of the cell membrane is destroyed, and LDH and CK could penetrate the membrane into the extracellular matrix (Gan et al., 2018). LDH and CK are two of the important diagnostic markers of myocardial injury. Our results showed that the four alkaloids could decrease the levels of LDH and CK significantly compared with the model group, indicating that they could protect H9c2 cells from hypoxia-induced membrane damage. In addition, the content of MDA and GSH as well as the activity of SOD can reflect the degree of oxidative damage and repair. To confirm the antioxidative activity of four alkaloids, the content of MDA and GSH and the activity of SOD were further detected. The results showed that the four alkaloids could improve the activity of the antioxidant defense enzyme SOD and the content of non-enzymatic antioxidant GSH and reduce the production of MDA. Therefore, they could relieve the oxidative stress-induced injury to a great extent and protect H9c2 cells against OGD-induced injury. Apoptosis, a form of programmed cell death, is regulated by genes in a physiological or pathological environment. Hoechst/PI staining and flow cytometry assay were used to analyze the morphological change and apoptosis rate of H9c2 cells. The results indicated that OGD treatment could induce H9c2 cell apoptosis, while the four alkaloids protected H9c2 cells against OGD-induced apoptosis effectively, suggesting that the cardioprotective effect of Menispermi Rhizoma was closely related to antiapoptotic effects. The expression of pro- and anti-apoptotic proteins was further verified by Western blotting. The results showed that the expression ratio of Bcl-2/Bax decreased, and the expression levels of Cyt-C and cleaved caspase 3 increased in the model group (*p* < 0.01), while the four alkaloids increased the expression ratio of Bcl-2/Bax and reduced the expression levels of Cyt-C and cleaved caspase 3. Through the KEGG enrichment analysis, it was found that the pathways of Menispermi Rhizoma on anti-MI mainly involve the PI3K/AKT and MAPK signaling pathways. Therefore, the key proteins (AKT1, MAPK1, EGFR, CASP3, and MAPK8) in the PI3K/AKT and MAPK pathways were selected to conduct molecular docking with the four active alkaloids. The results showed that acutumine, daurisoline, dauricoside, and 6-*O*-demethylmenisporphine had good binding activity with five key proteins. Therefore, these results suggested that Menispermi Rhizoma played an anti-MI role via the PI3K/AKT, MAPK, and apoptosis signaling pathways.

## 5 Conclusion

In this study, serum pharmacochemistry and network pharmacology were used to explore the effective components, therapeutic targets, and pharmacological mechanisms of Menispermi Rhizoma in the treatment of MI. From this network, we speculated the relationship between Menispermi Rhizoma and MI through multiple targets and several key signaling pathways. These results were partly confirmed by an OGD-induced H9c2 cardiomyocyte model and molecular docking experiments. Based on these combined findings, we concluded that the therapeutic effect of Menispermi Rhizoma on MI might be achieved by regulating the PI3K/AKT, MAPK, and apoptosis pathways. Herein, the active components of Menispermi Rhizoma in the treatment of MI mainly include acutumine, daurisoline, dauricoside, and 6-*O*-demethylmenisporphine. Meanwhile, Menispermi Rhizoma was found to show antioxidative and antiapoptotic effects on myocardial ischemic injury. Our findings elucidated the material basis and mechanism of Menispermi Rhizoma in treating MI, and provide insights for the further study of the cardiac protective effect of Menispermi Rhizoma. This study will provide a reference for further exploring the mechanism of Chinese herbal medicine in the treatment of diseases from a holistic perspective.

## Data Availability

The original contributions presented in the study are included in the article/Supplementary Material, further inquiries can be directed to the corresponding authors.

## Abbreviations

Bcl-2, B-cell lymphoma 2; Bax, Bcl-2-associated X protein; BCA, bicinchoninic acid; BPI, base peak intensity; Cyt-C, cytochrome c; CMC-Na, sodium carboxymethyl cellulose; CK, creatine kinase; ESI, electrospray ionization; GO, gene ontology; KEGG, Kyoto Encyclopedia of Genes and Genomes; GSH, glutathione; LDH, lactate dehydrogenase; MDA, malondialdehyde; MI, myocardial ischemia; MTT, 3-(4,5-dimethylthiazol-2-yl)-2,5-diphenyltetrazolium bromide; OGD, oxygen-glucose deprivation; PI3K/AKT, phosphatidylinositol-4,5-bisphosphate 3-kinase/protein kinase B; PBS, phosphate-buffered saline; PI, propidium iodide; SOD, superoxide dismutase; TCM, traditional Chinese medicine; UPLC-Q-TOF/MS, ultra-performance liquid chromatography quadrupole time-of-flight tandem mass spectrometry.
