# Supplementary material for: Integrated Serum Pharmacochemistry and Network Pharmacology Approach to Explore the Effective Components and Potential Mechanisms of Menispermi Rhizoma Against Myocardial Ischemia
Source: Front Chem. 2022 May 19;10:869972. doi: 10.3389/fchem.2022.869972 (PMC9160829; doi:10.3389/fchem.2022.869972)
Supplement: Supplementary file 1 [file DataSheet1.docx]

**[Supplementary materials](https://review.frontiersin.org/Document/DownloadSupplementaryMaterial?articleId=869972&userId=1220088&roleId=16)**

**Integrated serum pharmacochemistry and network pharmacology approach to explore the effective components and potential mechanisms of Menispermi Rhizoma against myocardial ischemia**

**Jinxia Wei^1*†^, Yingying Yu^2, 3†^, Yue Zhang^1†^, Lingzhi Li^2†^, Xia Li^2^, Jia Shao^4*^ and Yubo Li^1*^**

*^1^ School of Chinese Materia Medica, Tianjin University of Traditional Chinese Medicine, Tianjin, China*

*^2^ Department of Pharmacy, Logistical University of Chinese People's Armed Police, Tianjin, China*

*^3^Department of Health Service, Hunan Provincial Hospital of Chinese People's Armed Police, Changsha, China*

*^4^ Department of Pharmacy, Tianjin First Central Hospital, Tianjin, China*

*** Correspondence:**

E-mail address: syykdxwjx410@163.com (Jinxia Wei); houjiashao@sina.com (Jia Shao); [yaowufenxi001@sina.com](mailto:yaowufenxi001@sina.com) (Yubo Li)

**^†^**These authors contributed equally to this work

**Validation of compounds by *in vitro* experiments**

**1. Cell culture and treatment**

H9c2 cells were cultured in Dulbecco's modified Eagle medium (DMEM, Solarbio Co., Ltd, Beijing, China) with 10% fetal bovine serum (FBS, Lonsa Science SRL, Jiangsu, China) in a humidified incubator at 37 °C in 5% CO_2_. In the oxygen-glucose deprivation (OGD) model group, H9c2 cells were cultivated in the 94% N_2_-5% CO_2_-1% O_2_ incubator with oxygen-removed D-Hank’s solutions instead of DMEM and FBS.

**2. Cell proliferation assay**

For cell viability detection, H9c2 cells were seeded into 96-well plates and cultivated in cell culture medium. When cells confluence came up to 90%, the cells were grouped and treated as follows: the culture medium was removed from plates, and the synchronization was completed by serum-free DMEM infiltration for 12 h. After incubation, DMEM in the control group (normoxia) was replaced with fresh serum-free DMEM high-glucose medium, and then cells were cultured in the incubator (5% CO_2_, 37 °C) for 2 h. While for the OGD model group, the culture medium was removed and replaced by oxygen-removed D-Hank’s solutions, and then the cells were incubated in the hypoxia environment (94% N_2_-5% CO_2_-1% O_2_, 37 °C) for 2 h in a three-gas incubator. For drug intervention groups, including acutumine, daurisoline, dauricoside, 6-*O*-demethylmenisporphine, and verapamil, cell culture medium was replaced with the same volume of corresponding drug solution diluted in oxygen-removed D-Hank’s solution compared with the OGD model group, and then the cells were cultured in the same hypoxia environment as the model group for 2 h. 20 μL MTT solution (5 mg/mL) was added into the 96-wells and incubated for another 4 h at 37 °C. After discarding the supernatant, the crystallization of each well was dissolved with 150 μL dimethyl sulfoxide (DMSO). The absorbance was measured at 490 nm with a microplate reader (Model 680, Bio-Rad Laboratories, Inc). The mean optical density of 6 wells in each group was used to calculate relative cell viability in comparison with control group. The cell proliferation experiments were repeated for three times.

**3. Detection of myocardial enzyme (LDH and CK)**

LDH leakage and CK release serve as indicators of cardiomyocytes death or damage. The activities of LDH and CK were tested by colorimetric method with detection kits. H9c2 cells were seeded into 24-well plates at a concentration of 1 × 10^5^ cell/mL with 3 wells as a group. Then, the cells in each group were treated as the previous description in “Section 2”. Following the corresponding manufacturer’s instructions, the supernatant per well was collected to detect the LDH leakage and CK release by the assay kits. All measurements were performed in triplicate.

**4. Oxidative stress relative factors measurement**

Oxidative stress plays a crucial role in the development of myocardial injury. H9c2 cells were seeded into 6-well plates with 3 wells as a group. Then, the cells in each group were treated as the previous description in “Section 2”. The cells were washed with cold phosphate buffered saline (PBS) and collected into the Eppendorf tubes after centrifugation (300 ×g, 10 min), while the supernatant was aspirated off. In order to determine the intracellular content, cell disruption was performed in saline solution on ice bath with ultrasonic cell crushing apparatus (JY95-Ⅱ, Xinzhi Research Institute). The cell crushing procedure was set to 5 cycles with ultrasound for 5 s and rest for 30 s. Meanwhile, the measurement of intracellular protein concentration was necessary and BCA assay could quickly complete the test in 30 min. Finally, the measurements of SOD, GSH, and MDA were conducted by the assay kit following the manufacturer’s instructions.

**5. Cell apoptosis evaluation**

*Hoechst-33342/PI staining*: The H9c2 cells were seeded on the clean cover glass in 6-well plates. After undergoing OGD treatment, H9c2 cells were washed three times with ice-cold PBS. Then, the cells were dyed with fluorescent dyes. Briefly, 5 μL Hoechst-33342, 5 μL propidium iodide (PI) and 1 mL staining binding solution were added into every single well and the plates were incubated for 15 min in the dark at 4 °C. After being rinsed twice with cold PBS, whether cells were apoptosis or not was determined by capture under a fluorescent microscope.

*Flow cytometry assay*: Detection of H9c2 cells apoptosis rates was performed by flow cytometry with an Annexin V/PI apoptosis detection kit according to the manufacturer’s instructions. H9c2 cells were digested with EDTA-free trypsin, and then harvested and suspended in 100 μL binding buffer with 5 μL Annexin V and 5 μL PI. After incubation for 15 min in the dark, the percentage of apoptotic H9c2 cells was detected by flow cytometry (Beckman, USA).

**6. Western blotting analysis**

The expression levels of apoptosis-related proteins (Bax, Bcl-2, Cyt-C and cleaved caspase 3) were detected by western blotting. The protocol was performed following the procedure as the previous report (Shi et al., 2020). Briefly, the total protein from H9c2 cells were prepared with RIPA lysis buffer (CWBIO, Beijing, China) mixed with 1% PMSF and 1% phosphatase inhibitor, and then the supernatant was collected after centrifuging at 20 000 ×g for 10 min. The proteins concentration was detected by BCA assay, and adjusted to the same level by addition of distilled water. The protein samples were heated to denature at 100 °C for 15 min and eventually diluted with 5× loading buffer with bromocresol blue indicator. Then, the sample proteins of different groups were separated with SDS-PAGE gel (12%) and transferred to polyvinylidene difluoride (PVDF) membranes (Millipore, USA). After incubation for 1 h in 5% fat-free milk (w/v), the membranes were incubated with primary antibodies at 4 °C overnight, then followed by the incubation with secondary antibodies. Through the specific combination between primary antibody and protein, and the unspecific combination between primary antibody and secondary antibody

(HRP-conjugated rabbit/mouse IgG), the bindings were finally visualized with ECL chemiluminescence solution in Tanon 5200 software (Shanghai, China) and quantified by Image J 1.42q (National institution of health, USA). β-actin acted as an internal control.

**References**

Shi, C. F., Li, Z. C., Wu, Y. Q., Li, X., Li, Y. X., Wei, J. X., et al. (2020). Euscaphic acid and tormentic acid protect vascular endothelial cells against hypoxia-induced apoptosis via PI3K/AKT or ERK 1/2 signaling pathway. *Life Sci.* 252, 117666. doi:10.1016/j.lfs.2020.117666


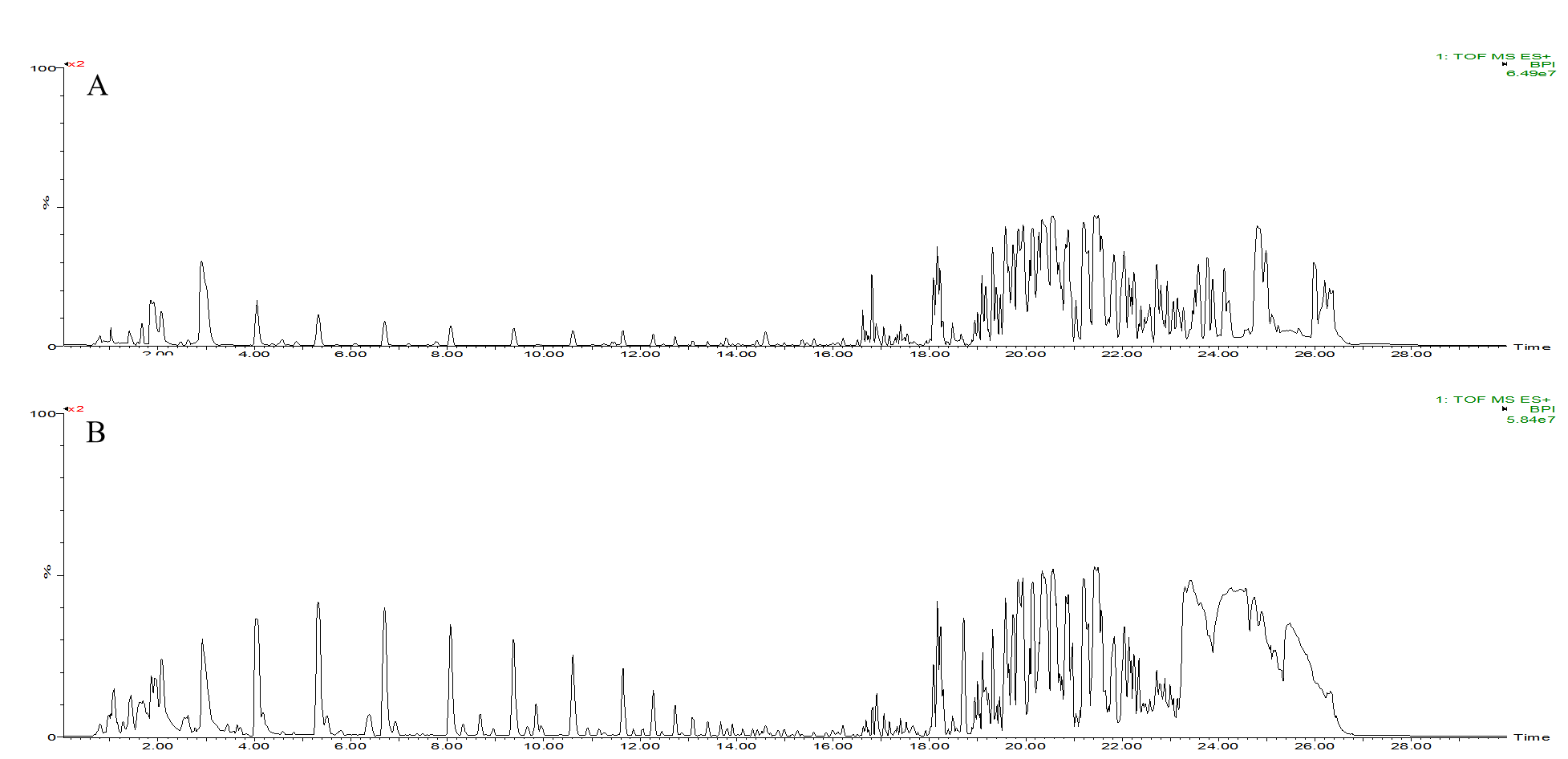


**Figure S1** BPI chromatograms of blank rat plasma (A) and actual plasma sample after oral administration of Menispermi Rhizoma extract (B) in the positive ion mode determined by UPLC-Q-TOF/MS.
